# Quantification, improvement, and harmonization of small lesion detection with state-of-the-art PET

**DOI:** 10.1007/s00259-017-3727-z

**Published:** 2017-07-08

**Authors:** Charlotte S. van der Vos, Daniëlle Koopman, Sjoerd Rijnsdorp, Albert J. Arends, Ronald Boellaard, Jorn A. van Dalen, Mark Lubberink, Antoon T. M. Willemsen, Eric P. Visser

**Affiliations:** 1 Department of Radiology and Nuclear Medicine, Radboud University Medical Centre, Nijmegen, The Netherlands; 2 MIRA Institute for Biomedical Technology and Technical Medicine, University of Twente, Enschede, The Netherlands; 3 Department of Nuclear Medicine, Isala Hospital, Zwolle, The Netherlands; 4 Department of Medical Physics, Catharina Hospital, Eindhoven, The Netherlands; 5 Department of Nuclear Medicine & Molecular Imaging, University of Groningen, University Medical Centre Groningen, Groningen, The Netherlands; 6 Department of Radiology and Nuclear Medicine, VU University Medical Center, Amsterdam, The Netherlands; 7 Department of Medical Physics, Isala, Zwolle, The Netherlands; 8 Department of Surgical Sciences, Uppsala University, Uppsala, Sweden; 9 Department of Medical Physics, Uppsala University Hospital, Uppsala, Sweden

**Keywords:** Time-of-flight, Point-spread-function, Digital PET, PET/MR, Lesion detectability, EARL

## Abstract

In recent years, there have been multiple advances in positron emission tomography/computed tomography (PET/CT) that improve cancer imaging. The present generation of PET/CT scanners introduces new hardware, software, and acquisition methods. This review describes these new developments, which include time-of-flight (TOF), point-spread-function (PSF), maximum-a-posteriori (MAP) based reconstruction, smaller voxels, respiratory gating, metal artefact reduction, and administration of quadratic weight-dependent ^18^F–fluorodeoxyglucose (FDG) activity. Also, hardware developments such as continuous bed motion (CBM), (digital) solid-state photodetectors and combined PET and magnetic resonance (MR) systems are explained. These novel techniques have a significant impact on cancer imaging, as they result in better image quality, improved small lesion detectability, and more accurate quantification of radiopharmaceutical uptake. This influences cancer diagnosis and staging, as well as therapy response monitoring and radiotherapy planning. Finally, the possible impact of these developments on the European Association of Nuclear Medicine (EANM) guidelines and EANM Research Ltd. (EARL) accreditation for FDG-PET/CT tumor imaging is discussed.

## Introduction

PET/CT is nowadays widely used in oncology and has become an essential multimodality imaging method that provides both anatomic and metabolic information [1, 2]. PET/CT imaging is important for the detection, localization, characterization, and staging of cancer [2]. However, the two main limitations of PET are the relatively low spatial resolution, which results in a partial-volume effect (PVE) affecting images both visually and quantitatively [3], and the generally low signal-to-noise ratio (SNR). The PVE limits the detection of small, low-contrast lesions (typically <2 cm), since they appear to be larger while their radiopharmaceutical uptake appears to be lower than the actual value, due to spill out of activity [4]. In addition, this also decreases the detection sensitivity itself when the signal-to-noise ratio of these lesions becomes too small. These effects are especially important when accurate quantification is needed. In recent years, there have been multiple advances in PET/CT that potentially improve cancer imaging and small lesion detection. In this article, these recent advances in PET/CT technology are explained. Also, the potential consequences of these developments for the EANM guidelines and EARL accreditation for FDG-PET imaging are discussed.

### New PET technologies and image reconstruction methods

In this section, an overview of several PET technological developments that took place during the last decade will be given, as well as a short description of their underlying principles. In particular, this review addresses TOF [5], PSF modeling [6], MAP-based reconstruction [7], smaller voxels [8], respiratory gating [9], metal artefact reduction [10], as well as hardware improvements like CBM [11], the development of solid-state photodetectors using digital photon counting technology [12] and the introduction of combined PET/MR imaging [13].

Our descriptions will be limited to those features that are currently available in commercial, clinical whole-body PET/CT, and PET/MR systems. Nevertheless, still newer developments are under way, and might enter the market within the coming years. Among these, the most important ones in our opinion, could be the following. New PET reconstruction methods for which PET attenuation correction by CT is not necessary [14]. This can reduce or avoid several artefacts (motion, metal) in the PET images, and leads to lowering of the radiation dose. Further, a substantial improvement of the TOF timing resolution (see next section) can be expected [5], thus improving image quality, reducing scan time, or reducing administered activity. Finally, scanners with very large axial FOV, such as the total body system proposed by Cherry et al. [15] could provide an even larger improvement of these parameters.

#### Time-of-flight

PET imaging is based on the detection of annihilation photons along a line-of-response (LOR). When the difference in arrival time between two annihilation photons is known, the location from which these photons originated can be determined. If this difference equals Δ*t*, the location of the annihilation event, with respect to the midpoint between the two detectors, is given by Δ*x = c* Δ*t/2*, where *c* is the speed of light (3 × 10^8^ m/s). This technique is called time-of-flight PET.

In 2006, the first commercial whole-body TOF-PET scanners were introduced. These PET scanners use lutetium oxyorthosilicate (LSO) or lutetium-yttrium oxyorthosilicate (LYSO) scintillators, which provide a timing accuracy of 350–550 ps, resulting in a localization accuracy of 5.3–8.3 cm. Table 1 shows vendor-specific timing and localization accuracy information. The spatial resolution of PET without TOF is already in the order of several millimeters. This indicates that TOF information will not directly lead to a higher spatial resolution. However, the incorporation of TOF information in the PET image reconstruction algorithm does provide images with a higher SNR, which improves the detection of small lesions with relatively low activity that would otherwise have been indistinguishable due to background noise. The SNR is approximated by SNR_TOF_ ≈ √(*D*/Δ*x)* ӿ SNR_non-TOF_ where *D* is the effective patient diameter [25]. Therefore, the effect of TOF is most pronounced in obese patients [5, 25, 26]. It has been shown that the SNR (as a property of the image) is proportional to the square root of the noise equivalent counts (NEC) [27], which is a property of the PET scanner. The increase in SNR is sometimes regarded as a gain in counts: a TOF image is equivalent to a non-TOF image obtained with a larger number of counts, where *D*/Δ*x* is called the gain factor. The sensitivity times this gain factor is sometimes called the effective sensitivity. In other words, the incorporation of TOF information increases the effective sensitivity. This can be used to provide better image quality and improved lesion detection, or to shorten the scan time while keeping the same image quality with better clinical workflow and added comfort for the patient, or finally to reduce radionuclide costs and reduce radiation dose to the patient and hospital personnel with the same scan time and image quality.

**Table 1:** PET/CT and PET/MR system specifications, including the availability and performance of recent developments such as TOF, PSF, respiratory gating, and metal artefact reduction. *Data from peer-reviewed publications. **Data from vendor. ***Data determined by authors (typical values measured during acceptance testing). Note about effective sensitivity: As explained in the section about time-of-flight, the gain factor by which the sensitivity is effectively increased by using TOF can be approximated as *D*/Δ*x*. However, most manufacturers (except Toshiba) do not exactly specify how they have calculated their effective sensitivity. When the effective sensitivity was not known, the authors calculated this by using the NEMA sensitivity and the TOF information with D = 20 cm

	Philips Vereos [16, 17]	Philips Ingenuity TF [18]	GE Discovery PET/CT 710 [12, 19]	GE Discovery IQ (five-ring system) [12, 20]	GE Discovery MI (four-ring system)	Siemens Biograph mCT Flow (TrueV) [11]	Toshiba Celesteion [21]	Mediso Anyscan	GE Signa [12, 22]	Siemens mMR [23, 24]
Patient port (cm)	70	70 Open view	70	70	70	78	88	70	60	60
MR	N/A	N/A	N/A	N/A	N/A	N/A	N/A	N/A	3 T	3 T
Patient scan range (cm)	190	190	200	200	200	198 FlowMotion: 195	179	235	188	200
Maximum patient weight (kg)	195	195	226	226	226	226	205	229	226	200
Crystal size (mm^3^)	4 × 4 × 22	4 × 4 × 22	4.2 × 6.3 × 25	6.3 × 6.3 × 30	3.95 × 5.3 × 25	4 × 4 × 20	4 × 4 × 12	3.9 × 3.9 × 20	4.0 × 5.3 × 25	4 × 4 × 20
Photodetector	SiPM	PMT	PMT	PMT	SiPM	PMT	PMT	PMT	SiPM	APD
Axial FOV (cm)	16.4	18	15.7	26	20	22.1	19.6	23	25	25.8
Scintillation detector material	LYSO	LYSO	LYSO	BGO	LYSO	LSO	LYSO	LYSO	LYSO	LSO
NEMA system sensitivity at center (kcps/MBq)	5.7*	7.4*	7.1***	22.8*	13.5**	9.6*	>3.6**	9.1**	22.9*	13.3*
Effective sensitivity (kcps/MBq)	24.1*	>18.8**	17.3*	22.8*	46.6**	25.5**	≥10.8**	?	76.3*	13.3*
NEMA radial resolution (FWHM) @ 10 cm	4.6*	5.3*	5.1* (average radial and tangential)	5.6*	4.5** (average radial and tangential)	5.2*	<5.1** (average radial and tangential)	4.9** (average radial and tangential)	5.8*	5.2*
NEMA tangential resolution (FWHM) @ 10 cm	4.2*	5.0*	5.1* (average radial and tangential)	5.1*	4.5** (average radial and tangential)	4.7*	<5.1** (average radial and tangential)	4.9** (average radial and tangential)	4.4*	4.8*
NEMA axial resolution (FWHM) @ 1 cm and 10 cm	3.8/4.0*	4.7/5.2*	4.8/5.6*	4.8/4.8*	4.8/4.7**	4.3/5.9*	<5.0/<5.4**	4.2/5.1**	5.4/6.8*	4.3/6.6*
Peak NECR (kcps) @ kBq/ml	171@50*	124@20*	144@29***	124@9*	180@20**	185@29*	61 +/- 10**	150**	215@18*	196@24*
TOF timing resolution (ps)	345**	495**	544*	N/A	385**	540**	<450**	?	<400*	N/A
TOF localization accuracy (cm)	5.2**	7.4**	8.2*	N/A	5.8**	8.1**	<6.8**	?	6.0*	N/A
PSF	y (PSF)	y (PSF)	y (SharpIR)	y (SharpIR)	y (SharpIR)	y (HD)	y	?	y (SharpIR)	y (HD)
Respiratory gating	Phase-based	Phase-based	Phase-based (Q.Static/Q.Freeze)	Phase-based (Q.Static)	Phase-based (Q.Static/Q.Freeze)	Amplitude-based (HD•Chest)	Phase-based	?	Phase-based (Q.Static/Q.Freeze)	Amplitude-based (HD•Chest)
Metal artefact reduction	O-MAR	O-MAR	Smart MAR		Smart MAR	iMAR	SEMAR	?		WARP

#### Point-spread-function modeling

Iterative image reconstruction methods use a system matrix that couples the coincidence counts along each LOR to the activity in the different voxels. In principle, this matrix takes into account all processes that influence the measured counts along each LOR. Among these are resolution degrading effects such as positron range, photon non-colinearity, and detector-related effects, including crystal widths, inter-crystal scattering, and inter-crystal penetration (depth of interaction effects). Resolution modeling or PSF modeling takes into account these effects during image reconstruction [6]. However, PSF modeling can also be applied as a post-reconstruction deconvolution [28]. The first method has been implemented by Siemens (HD) and GE (SharpIR), while the second method is used by Philips, as can be seen in Table 1.

It has been demonstrated that PSF modeling in PET reconstructions leads to higher and more uniform spatial resolution over the transaxial FOV [29–31]. Special attention should be given to some pitfalls, noise and Gibbs artefacts can be amplified [32]. However, for noise, this depends on its definition. As explained by Alessio et al. [33], PSF modeling can reduce noise when it is defined as intensity variation on a voxel-to-voxel basis, but may increase the ensemble standard deviation of mean lesion uptake. Also, spatially correlated noisy patterns can be introduced, especially for low count statistics [34].

**Fig. 1:**
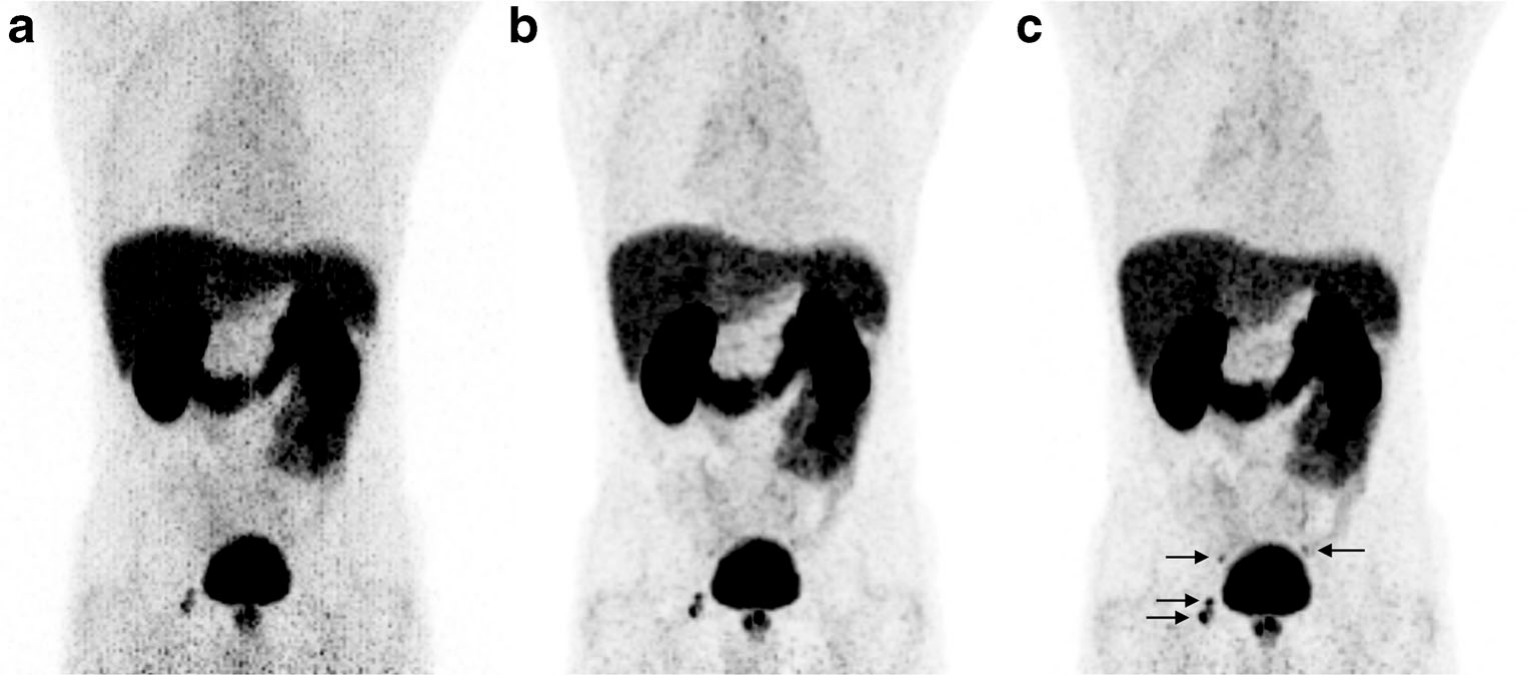
^68^Ga-labeled prostate-specific membrane antigen (PSMA) maximum intensity projection PET images (mCT, Siemens) of a patient with metastasized prostate cancer. PSMA uptake is visible in the prostate and four metastases (two lesions in the acetabulum (*right*), and two para-iliac lymph nodes (*left and right*)). All images were reconstructed with a transaxial matrix size of 256 × 256, pixel size of 3.1 × 3.1 mm^2^. (**a**) PET reconstruction without PSF modeling and without TOF, (**b**) PET reconstruction with PSF modeling and without TOF, and (**c**) a PET reconstruction with both PSF modeling and TOF (data are from Radboudumc, Nijmegen, The Netherlands)

An example of a clinical PET scan demonstrating the impact of TOF and PSF is shown in Fig. 1. It is interesting to note that although PSF modeling was developed and tested mainly for ^18^F–FDG imaging, it clearly also enhances small lesion detectability using ^68^Ga-based tracers. Apparently, this is not hampered by the higher positron energy and larger range for ^68^Ga versus ^18^F.

#### Bayesian penalized likelihood

When using conventional iterative reconstruction algorithms based on maximum likelihood estimation maximization (MLEM) such as ordered subset expectation maximization (OSEM), the quantitative accuracy of the resulting images improves (the standardized uptake values (SUVs) of lesions increase) when the number of iterations is increased. However, image noise levels also increase with each iteration, hampering visual small lesion detection. As a compromise, some bias (underestimation of SUV in smaller lesions) is allowed in the reconstructed images in return for reduced noise levels, by stopping the iterative process after a limited number of iterations, or by applying post reconstruction spatial smoothing [35].

**Fig. 2:**
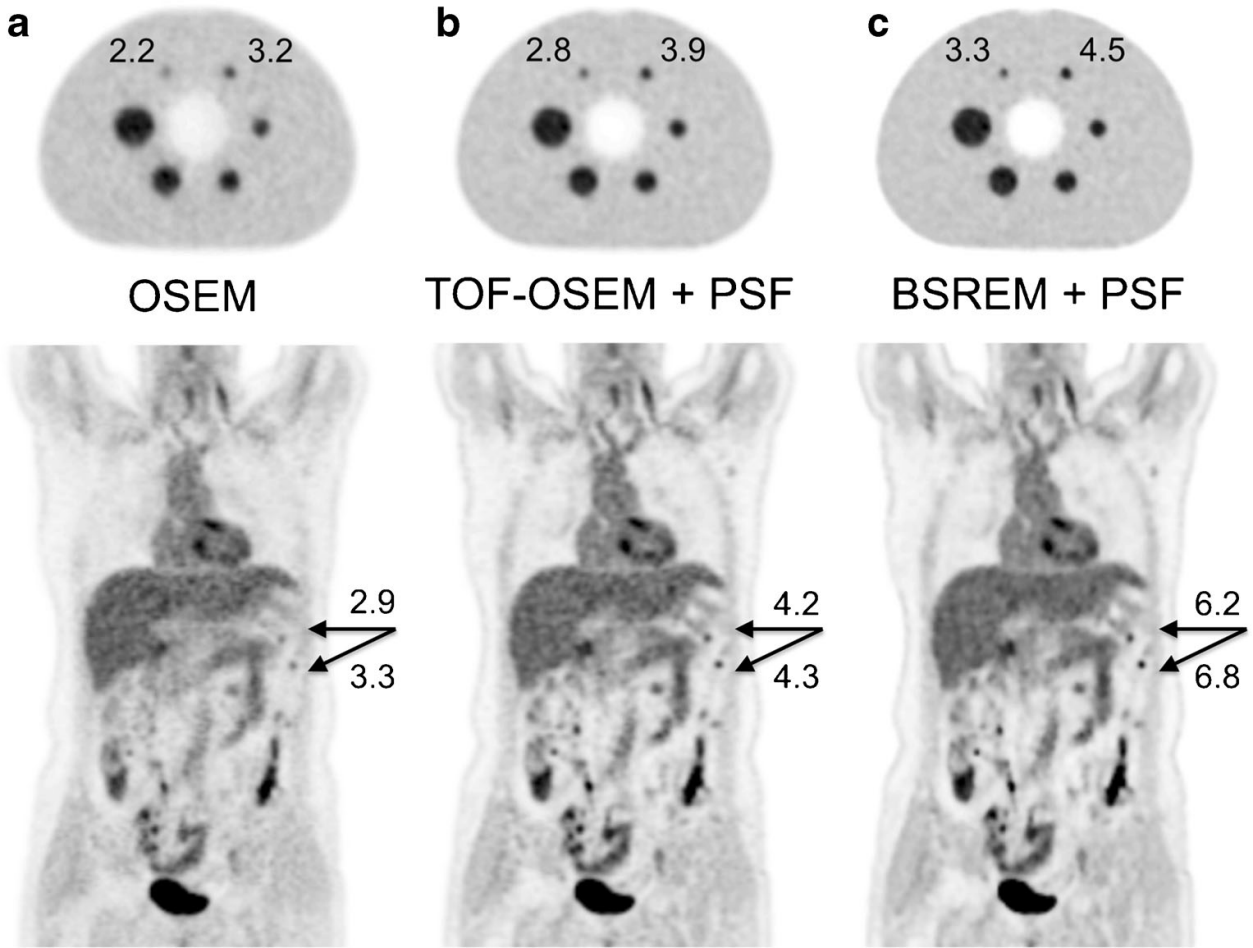
*Top row*: images of a 100 Mcounts acquisition of the NEMA image quality phantom (sphere-to-background activity concentration ratio 4:1). Measured sphere-to-background ratios (hottest pixel) are given for the two smallest spheres. *Bottom row*: ^18^F–FDG PET images (four-ring Discovery MI, GE) of a patient with ovarian cancer with peritoneal carcinomatosis, (**a**) reconstructed using OSEM, (**b**) TOF-OSEM with PSF modeling, and (**c**) block-sequential regularized expectation maximization (BSREM; Q.clear) with PSF modeling and a beta-value of 400. SUV_max_ [g/cm^3^] is given for the two lesions. Note the much better recovery in the small lesions when adding TOF and PSF, with further improvement for BSREM, optimized for BPL. The beta value in the BSREM reconstruction was chosen to result in similar background variability in the BSREM and TOF-OSEM images of the NEMA phantom (data are from Uppsala University Hospital, Uppsala, Sweden)

Bayesian methods are applied in PET image reconstructions to further improve the quality of reconstructed images by taking advantage of prior knowledge of the image, e.g., non-negativity of the tracer concentration, limited variation between neighboring voxels (while preserving real edges), or anatomical information for example from CT. The Bayesian penalized likelihood technique (BPL) or MAP algorithm (for instance as incorporated in Q.Clear (GE) [7]) allows effective convergence of image accuracy while suppressing noise, by using a penalty function [7, 36]. With every iteration, the outcomes with lower variation between neighboring voxels are slightly favored over noisier ones. The strength of this penalty term is chosen to match the procedure type. A substantial number of iterations (typically 25) warrants convergence without amplifying noise, resulting in improved image quality and increased SUV, particularly in small lesions when compared with reconstruction techniques without using MAP [7, 35, 37]. An example is given in Fig. 2.

#### Small voxel reconstruction

**Fig. 3:**
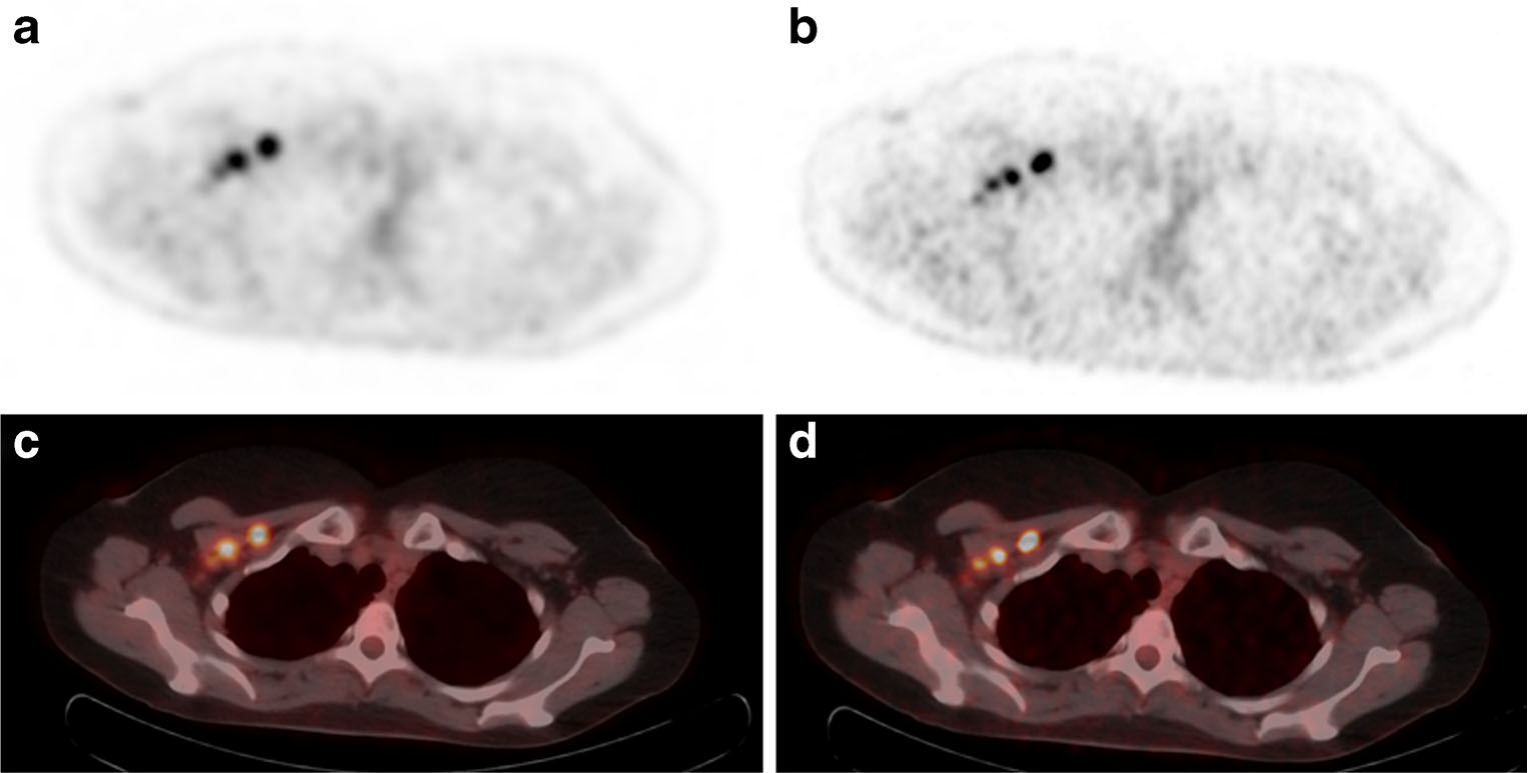
^18^F–FDG PET/CT images (Ingenuity TF, Philips) of a patient with metastasized breast cancer. The reconstructions were made without PSF modeling, but with TOF. (**a**, **c**) A standard 4 × 4 × 4 mm^3^ voxel reconstruction and (**b**, **d**) a small 2 × 2 × 2 mm^3^ voxel reconstruction. On the small-voxel images, there is an improved visualization of axillary lymph nodes, with an increase of SUV_max_ of more than 65% for the small lymph nodes (data are from Isala Hospital, Zwolle, The Netherlands)

In current practice, the image voxel size for whole-body FDG-PET scans is typically around 4 × 4 × 4 mm^3^ [18, 38, 39], which is in the order of the NEMA spatial resolution of the PET scanner [40], defined as the full width at half and tenth maximum (FWHM/FWTM) of a point source when reconstructed using filtered back-projection without any corrections. Recent studies demonstrated that the use of smaller voxels and corresponding larger matrices, in combination with TOF-PET/CT systems, improves the detection of small lesions [8, 41–43]. Li et al. [41] demonstrated that using a 400 × 400 matrix (2 × 2 mm^2^) resulted in more detected lymph nodes and a better visual image quality, as compared to a 200 × 200 matrix (4.1 × 4.1 mm^2^). Furthermore, Koopman et al. [8] showed that the use of 2 × 2 × 2 mm^3^ instead of 4 × 4 × 4 mm^3^ voxels was preferred by physicians, based on rankings including lesion sharpness, lesion contrast, and diagnostic confidence. Moreover, the use of 2 × 2 × 2 mm^3^ voxels resulted in an increase in SUV_mean_, SUV_max_, and SNR for small lesions (<11 mm) in patients. This is also demonstrated in Fig. 3. Additionally, they found that the contrast recovery coefficients (as defined in their paper) for phantom spheres were more accurate using 2 × 2 × 2 mm^3^ voxels [8].

A drawback of the use of small voxels is an increase of noise in the PET images as smaller voxels imply fewer counts per voxel [8]. These higher noise levels may result in more false-positive findings [44].

#### Respiratory gating

Respiratory motion causes blurring of lesions in the thorax and upper abdomen, and can cause additional artefacts because of an inaccurate attenuation correction due to a mismatch between PET and CT [45]. This results in a lower detectability of tumors, inaccurate SUVs, and sub-optimal radiotherapy treatment planning [46, 47]. Respiratory gating can be used to create an essentially motion-free PET image. There are two methods that are most common. For the first method, the respiration of the patient is tracked and only a part of the PET data is used to reconstruct a motion-free image. For the second method, the respiration is also tracked, but all PET data is used to reconstruct a motion-free image by translating gated images of the different respiratory phases. In recent years, several respiratory gating methods have been developed for PET imaging [46, 48]. For the first method, to maintain image quality, respiratory gating requires a longer scan time and/or a higher injected activity. Therefore, respiratory gating is nowadays not routinely used for diagnostic imaging [49, 50]. However, it is more commonly applied for radiotherapy planning, where an accurate delineation and quantification is even more important [51–53].

**Fig. 4:**
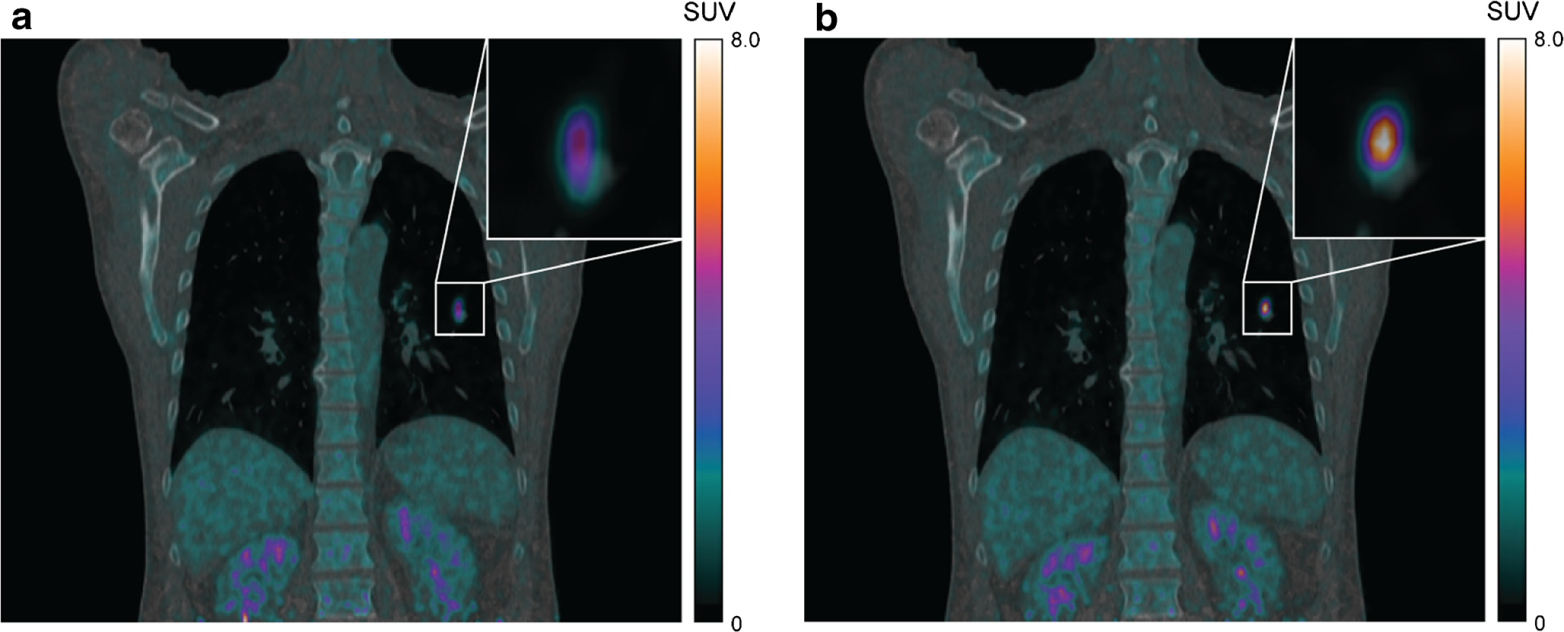
^18^F–FDG PET/CT images (mCT, Siemens) of a patient with a non-small cell lung cancer lesion in the left lower lobe. (**a**) Non-gated and (**b**) an essentially motion-free image (HD•Chest). Both PET images have been reconstructed with a matrix size of 400 × 400, pixel size of 2 × 2 mm^2^, with PSF modeling and TOF. For the non-gated images, the first 35% (126 s) of the acquired data was used for image reconstruction, resulting in an equal number of acquired true coincidences as the gated image. There is a considerable increase in SUV_mean_ of 70% and a decrease in volume of 80%. Images have been reproduced from [46]

Different vendors offer different respiratory gating methods. Philips, GE, and Toshiba use a phase-based gating method [54]. Siemens also allows phase-based gating, but in addition offers an amplitude-based optimal gating method, called HD•Chest. With this method only PET data collected from the respiratory amplitude range with the least amount of motion are used [9, 46]. GE also introduced Q.Freeze, which should only be used for diagnostic purposes. Q.Freeze is a phase-based gating method in combination with a non-rigid translation of the other phases, so all collected data are used for the final motion free image [48]. An example of the impact of respiratory gating on a PET image is shown in Fig. 4.

#### Metal artefact reduction

Metal artefact reduction is a standard tool in stand-alone CT systems and different methods are well described in the literature [55]. However in PET/CT, reduction of metal artefacts is relatively new, not commonly implemented, and little research has been performed on the impact of CT metal artefacts on PET imaging. Artefacts on CT images can influence the PET reconstruction, as CT data are used for PET attenuation correction. If the region of interest is located near the implant, the metal not only distorts the CT image but also influences the quantification of radiotracer uptake and can reduce the image quality and interpreter confidence [10, 56]. Metal artefact reduction is important for diagnosis [57] and therapy planning [58] in head and neck cancer, and it can improve the image quality of ^68^Ga-PSMA PET studies for metastasis detection in patients with one or two hip prostheses [10, 59].

**Fig. 5:**
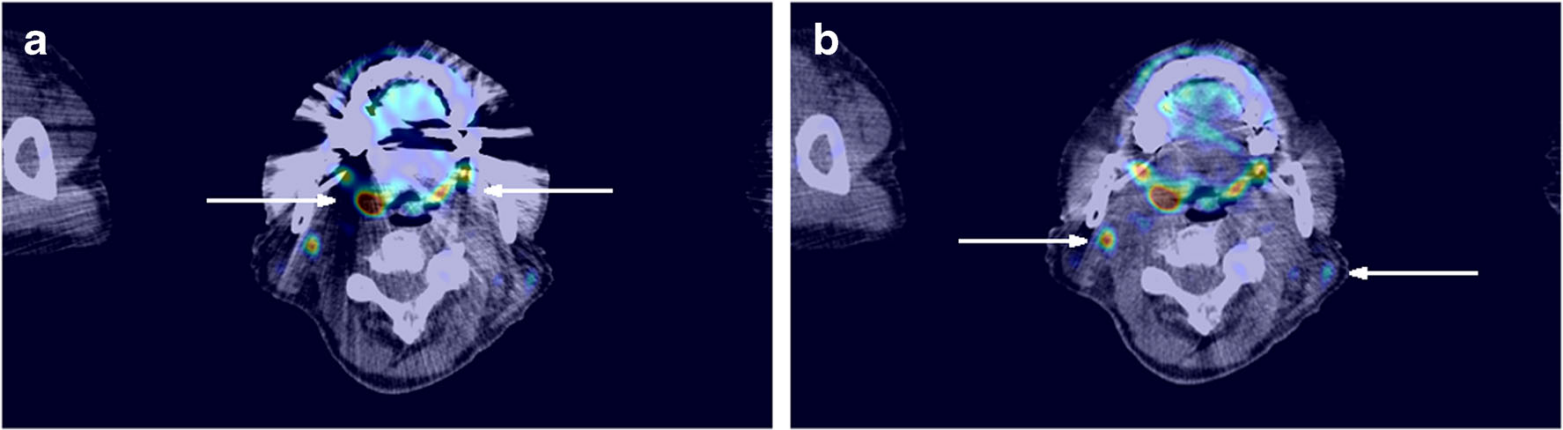
^18^F–FDG PET/CT images (mCT, Siemens) of a patient with uptake in the palatine tonsils (*arrows* in **a**), and ^18^F–FDG-avid lymph nodes (*arrows* in **b**). Both PET images have been reconstructed with a matrix size of 200 × 200, pixel size of 4 × 4 mm^2^, with PSF modeling and TOF. The metal artefact is visible on the (**a**) standard PET/CT reconstruction, while the (**b**) PET/CT reconstruction with metal artefact reduction (iMAR) shows fewer CT artefacts. There is an SUV_mean_ increase from 2.5 to 2.8 g/cm^3^ when iMAR is used for the tonsil. Images have been reproduced from [10]

Recently, iterative metal artefact reduction was introduced for some PET/CT scanners. Siemens introduced the iMAR algorithm [10], Philips introduced O-MAR and Toshiba SEMAR. It is expected that these algorithms result in an improved quantification and interpretation of the PET image near metal implants. An example is shown in Fig. 5.

#### Continuous bed motion

Due to the limited axial FOV of PET scanners, more than one bed position is generally needed to cover the section of the body that needs to be imaged. Since the sensitivity decreases toward the edges of the axial FOV, these bed positions are chosen to partly overlap to improve the uniformity in sensitivity along the axial direction [60]. Recently, CBM acquisition was introduced by Siemens (FlowMotion). The PET scanner shows similar performance compared to its predecessor system with discrete bed positions. The image quality was also similar for both techniques, with the exception of slightly increased noise levels for the planes at the edges of the outer bed positions in the standard acquisition [11, 61].

However, an advantage of the CBM technology is that the scan range can be selected without being restricted to a discrete number of bed positions, thus on average saving scan time by using a shorter scan range [62]. CBM could result in less CT radiation exposure due to this shorter range [62]. Finally, it has been stated that patients prefer the more fluent scanning of the CBM method over the more abrupt movements using discrete bed positions [61, 62].

### Solid-state and digital PET

Recently, three vendors introduced PET scanners based on solid-state photodetectors, replacing the conventional photomultiplier tubes (PMTs). Siemens introduced their mMR PET/MR scanner that uses avalanche photodiodes (APD), which can operate in a magnetic field, thus offering the possibility of constructing an integrated PET/MR scanner. GE introduced their Signa PET/MR scanner using silicon photomultipliers (SiPM), which can also operate in a magnetic field. Philips introduced the Vereos PET/CT scanner based on SiPMs with digital readout, and GE released their Discovery MI PET/CT scanner, also based on SiPMs with digital readout.

In case of the digital PET scanner from Philips, the digital SiPMs are capable of detecting and processing single scintillation photons because their elements match the size of the scintillator crystal elements and they incorporate electronics to achieve a one-to-one relation between the scintillator crystal elements and the digital photomultipliers [63–65]. In terms of system performance, this design results in an improved spatial and timing resolution and relatively high maximum count rates. In case of the Discovery MI scanner (GE), 12 crystals (4 × 3) are coupled to an array of SiPMs (3 × 2), much like the block design of analogue PMT-based scanners. This reduces count-rate capability and spatial resolution compared to one-to-one coupling of crystals and SiPMs, but improves sensitivity.

**Fig. 6:**
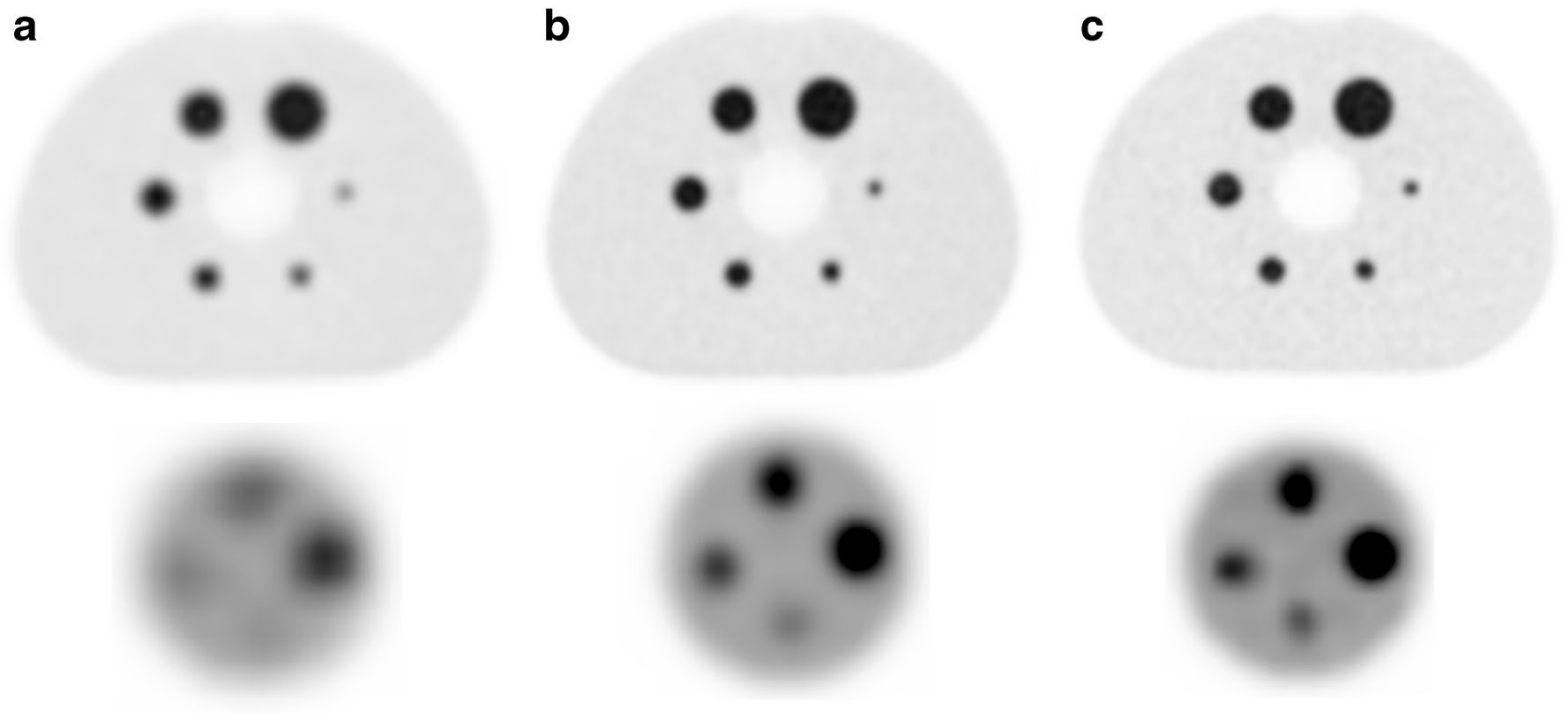
PET images of a NEMA phantom (sphere diameters 10–37 mm) and micro phantom (sphere diameters 4-8 mm), filled with 20 and 2 kBq/ml FDG in the spheres and the background, respectively. Data were acquired on an analogue, PMT-based PET (Ingenuity TF, Philips) and a digital SiPM-based PET (Vereos, Philips). (**a**) Images of the analogue PET that fulfils EARL requirements. (**b**) Images of the analogue PET using 2 × 2 × 2 mm^3^ voxel reconstruction. (**c**) Images of a digital PET using a 2 × 2 × 2 mm^3^ voxel reconstruction (data are from Isala Hospital, Zwolle, The Netherlands)

Based on phantom and patient studies that were recently performed on a digital PET system [16, 66, 67], it is expected that digital PET can provide a higher image quality and/or allow for a lower radiopharmaceutical dose and improved small lesion detection for oncology scans, as compared to an analogue PET system with PMTs. Figure 6 shows PET images of an analogue, PMT-based system and a digital PET system, of a NEMA image quality phantom (sphere diameters 10–37 mm) and a micro hollow sphere phantom (sphere diameters 4–8 mm). The reconstructed images demonstrate that image quality and small object detection improve using reconstruction settings with small voxels, on both the analogue and the digital PET. Furthermore, there is a higher contrast of the smallest spheres on the digital PET images as compared to the analogue PMT-based PET. Nguyen et al. [68] reported their initial experience in cancer patients with a prototype digital PET scanner compared to an analogue PET system with PMTs. They found a better image quality, diagnostic confidence, and accuracy with their digital PET.

### Hybrid PET/MR imaging

During the development of hybrid PET/MR systems, two major challenges needed to be overcome. First of all, conventional PET photodetectors are based on PMTs that cannot be operated in the high magnetic field of an MR scanner and are too large to allow placement inside an MR body coil whilst still leaving a sufficiently large patient opening. Integrated PET/MR was achieved using (analogue) APDs or SiPMs for conversion of the light produced by the scintillator crystals. In addition to their ability to function properly in a magnetic field, both APDs and SiPMs are much smaller than traditional PMTs, allowing for detector rings of about 5 cm thickness inside a 70-cm MR bore, leaving a 60-cm patient port diameter. An advantage of SiPMs compared to APDs is that SiPMs allow for TOF, whereas APDs, due to their timing resolution of about 2000 ps, do not. Specifications of the PET components for two fully integrated PET/MR systems are given in Table 1.

The second major challenge of quantitative PET with PET/MR, is that the PET attenuation correction needs to be derived from MR images, which essentially provide proton density rather than attenuation coefficients. Most PET/MR systems employ a dedicated (fast) MR sequence, followed by segmentations or tissue classification of the resulting MR image and assigning a priori known attenuation coefficients to a limited number of segmentation or tissue classes. This approach has several limitations. First of all, bone tissue is typically not included in this process and its attenuation is assumed to be equivalent to soft tissue attenuation. Secondly, lungs are segmented and assigned a uniform attenuation coefficient. Thirdly, the patient couch, fixation devices, and the coils used for MR image acquisition are not detected by the MR scanner and dedicated predefined attenuation templates need to be added to the attenuation image to compensate for them. Fourthly, the MR FOV is typically smaller than that of the PET scanner and truncation of the MR image in the transaxial direction is often observed, resulting in incomplete attenuation coefficient images and thus incorrect attenuation correction of the PET data. For most of the limitations indicated above, solutions have been proposed but not all of them are yet routinely available on all systems. For example ultra-short echo time (UTE) or zero echo time (ZTE) MR can be used to visualize bone and has only recently been introduced for brain PET/MR [69]. Another approach would be the use of CT-based templates which are registered onto the patients MR images and finally combined and processed to generate patient-specific attenuation images [69]. MR truncation artefacts in the attenuation images can be solved by first performing a PET reconstruction without attenuation, then derive the outer contour of the patient from this image and assign soft tissue attenuation to the tissues missed in the MR image [69]. However, advanced reconstruction methods, such as maximum likelihood of activity and attenuation (MLAA), might also be used to correct for MR truncation or otherwise incorrect attenuation maps [70–72]. A more complete overview of current PET/MR technologies, opportunities and challenges can be found in a review by Quick and Boellaard [73].

### Possible future implications of technological developments on imaging guidelines and applications

To date, most of the new technologies that were discussed in this paper are not yet widely spread in clinical practice. However, several of these, such as digital photodetector technology, PET/MR and novel PET reconstruction methods will become more available. We expect that they will be increasingly clinically used in the next decade and will have a large impact on image quality, lesion detection, and quantification in cancer PET imaging. These new technological developments thus provide a technology push for the evolution of new standards and imaging guidelines.

#### Imaging guidelines and quantitative standards

The EANM guidelines for FDG-PET/CT tumor imaging and the associated PET/CT system accreditation program run by EARL aim to harmonize the use of FDG-PET/CT in oncology as a quantitative imaging biomarker in multicenter studies [39]. To date, the EANM/EARL standard is based on the technological status for the majority of the installed PET/CT systems. In order to allow sites to benefit from the advantages of the new technologies described, two different PET reconstructions could be made: one optimized for visual interpretation and another meeting international quantitative standards [39, 74–76]. With the introduction of new acquisition and reconstruction techniques in the latest scanners from multiple vendors, and assuming that the availability and presence of PET scanners using older technology will decrease, it is expected that these technologies will become widely spread during the next 5 to 10 years. Consequently, EARL standards will need to be updated over time and the implication of new technologies on harmonized quantitative performance is presently being explored by EARL as discussed in more detail elsewhere in this supplement issue [77].

#### New applications facilitated by new technologies

The improved image quality can be used to adjust administered activity and/or scan duration. In 2013, de Groot et al. [78] published an optimized FDG-activity regimen, which is based on a quadratic relation between FDG-activity and patient’s body weight. They demonstrated that when using a quadratic administration regimen, the image quality (in terms of SNR in the liver) remains constant for patients with various body masses. This FDG-activity regimen has been mentioned as an alternative to the linear regimen in the second version of the EANM guidelines for FDG-PET tumor imaging [39]. Recently, a technical note was published by Koopman et al. [79] describing how to derive an FDG-activity formula, taking into account both EANM guidelines [39, 80] and a quadratic relation between FDG-activity and patient’s body weight. Their equation can be applied for all PET/CT systems, regardless of their technological status. A drawback of the quadratic administration of FDG-activity is that it requires a high amount of FDG-activity in obese patients. Alternatively, a quadratic-dependent duration of the PET scan could be implemented in these cases.

An example of a new application of PET/CT that has been facilitated by the recent developments in PET/CT technology is the use of ^90^Y–PET/CT imaging in patients with liver metastasis who were treated by selective internal radiation therapy (SIRT). ^90^Y is a radionuclide with a very small positron fraction (31.9 × 10^−6^) and therefore it is challenging to use it for PET imaging [81]. However, several studies have recently compared Bremstrahlung ^90^Y–single-photon emission computed tomography (SPECT)/CT with ^90^Y–PET/CT and demonstrated that ^90^Y–PET/CT scans using state-of-the-art TOF-PET systems provide a higher image quality with improved lesion detection and more accurate quantification and dosimetry [82–86].

Furthermore, the recent developments in PET/CT technology facilitate the use of low-count-rate PET studies such as imaging with ^124^I, which is performed in the follow-up of thyroid cancer. In general, the image quality for the ^124^I–PET scan is poor due to the complex decay scheme and especially the emission of prompt gamma rays with an energy of 602.7 keV, well within the standard energy window of a PET scanner. Furthermore, even higher energy gammas are present, which can downscatter into the energy window, or increase the dead time. For such a radionuclide, TOF results in a better SNR for the same number of counts [5, 87]. It is expected that recent developments in PET/CT technology, combined with a careful application of correction methods for the prompt gammas [88], further facilitate the use of ^124^I–PET/ CT [89] (or tracers labeled with other radionuclides such as ^89^Zr [90]) with an improved image quality and a more accurate quantification [91].

## Conclusions

In recent years, the development of PET/CT scanners has mainly focused on improved small lesion detection. The introduction of TOF, PSF modeling, and smaller voxels were the main reasons for this improvement. Also, an increased axial length increased the sensitivity of the scanner [60], while the spatial resolution was improved by reducing the size of the scintillator crystal element and by using smaller voxels [60]. Other reconstruction techniques have been developed for specific problems, such as respiratory gating and metal artefact reduction. Together, all these advancements made it possible to improve the quality and quantification of PET/CT images and optimize radiation dose and scan time.

The increase in effective sensitivity and improved spatial resolution led to an improved visibility of small lesions, which is not only important for detection of lesions and metastases in ^18^F–FDG-PET/CT scans, but also for other tracers, for instance the use of ^68^Ga-PSMA for the detection of (lymph node) metastases in patients with prostate cancer, ^89^Zr-MAb immunoPET studies, ^90^Y imaging for patients who are treated for liver metastasis, or ^124^I imaging for follow-up of thyroid cancer. This implies that PET/CT is nowadays not only used for detection and identification of lesions but has also been increasingly implemented for radiotherapy planning and therapy response monitoring [39]. For these applications, an accurate quantification and repeatability/reproducibility is of the utmost importance. The ongoing improvements discussed in this paper can contribute to this.
